# Risk factors for hospital-acquired pneumonia in hip fracture patients: A systematic review and meta-analysis: Retraction

**DOI:** 10.1097/MD.0000000000037907

**Published:** 2024-03-29

The article “Risk factors for hospital-acquired pneumonia in hip fracture patients: A systematic review and meta-analysis”^1^, which published in Volume 103, Issue 10 of *Medicine*, is being retracted due to duplicate publication.^2^ The manuscript was submitted to *Medicine* on June 19, 2023, nearly two months after being submitted to *BMC Musculoskeletal Disorders,* unbeknownst to the Editorial Office and underwent peer review. It was accepted for publication on October 3, 2013, at which time it was run through our iThenticate plagiarism software, and the duplication was not detected. Due to a lapse in communication, the article proceeded through production as usual and was published on March 8. The authors notified the Publisher of the duplicate publication and agreed to the retraction.
